# Is venous blood drawn from femoral access adequate to estimate the central venous oxygen saturation and arterial lactate levels in critically ill patients?

**DOI:** 10.5935/0103-507X.20150058

**Published:** 2015

**Authors:** Yara Nishiyama Marti, Flávio Geraldo Rezende de Freitas, Rodrigo Palácio de Azevedo, Milena Leão, Antônio Tonete Bafi, Flavia Ribeiro Machado

**Affiliations:** 1 Departament of Anesthesiology, Pain and Intensive Care, Universidade Federal de São Paulo - São Paulo (SP), Brazil.

**Keywords:** Femoral vein/physiology, Lactates, Oxygen consumption/physiology, Central venous pressure/physiology

## Abstract

**Objectives:**

The purpose of this study was to test if venous blood drawn from femoral access can be used to estimate the central venous oxygen saturation and arterial lactate levels in critically ill patients.

**Methods:**

Bland-Altman analysis and Spearman correlations were used to compare the femoral venous oxygen saturation and central venous oxygen saturation as well as arterial lactate levels and femoral lactate. A pre-specified subgroup analysis was conducted in patients with signs of hypoperfusion. In addition, the clinical agreement was also investigated.

**Results:**

Blood samples were obtained in 26 patients. In 107 paired samples, there was a moderate correlation (r = 0.686, p < 0.0001) between the central venous oxygen saturation and femoral venous oxygen saturation with a bias of 8.24 ± 10.44 (95% limits of agreement: -12.23 to 28.70). In 102 paired samples, there was a strong correlation between the arterial lactate levels and femoral lactate levels (r = 0.972, p < 0.001) with a bias of -2.71 ± 9.86 (95% limits of agreement: -22.03 to 16.61). The presence of hypoperfusion did not significantly change these results. The clinical agreement for venous saturation was inadequate, with different therapeutic decisions in 22.4% of the situation; for lactate, this was the case only in 5.2% of the situations.

**Conclusion:**

Femoral venous oxygen saturation should not be used as a surrogate of central venous oxygen saturation. However, femoral lactate levels can be used in clinical practice, albeit with caution.

## Abstract (pt)

**Objetivos:**

Testar se amostras de sangue venoso coletadas do acesso femoral podem ser utilizadas para estimar a saturação venosa central de oxigênio e os níveis de lactato arterial em pacientes graves.

**Métodos:**

Foram utilizadas a análise de Bland-Altman e correlações de Spearman para comparar a saturação venosa femoral de oxigênio e a saturação venosa central de oxigênio, assim como os níveis de lactato arterial e femoral. Foi conduzida uma análise predeterminada de subgrupos nos pacientes com sinais de hipoperfusão. Além disso, foi também investigada a concordância clínica.

**Resultados:**

Foram obtidas amostras sanguíneas de 26 pacientes. Em 107 amostras pareadas, observou-se correlação moderada (r = 0,686; p < 0,0001) entre a saturação venosa central de oxigênio e a saturação venosa femoral de oxigênio, com um viés de 8,24 ± 10,44 (limites de concordância de 95%: -12,23 a 28,70). Em 102 amostras pareadas, houve forte correlação entre os níveis arteriais de lactato e os níveis de lactato femoral (r = 0,72, p < 0,001) com um viés de -2,71 ± 9,86 (limites de concordância de 95%: -22,3 a 16,61). A presença de hipoperfusão não modificou de forma significante os resultados. A concordância clínica para saturação venosa foi inadequada, com diferentes decisões terapêuticas em 22,4% das situações; para o lactato, isto ocorreu em apenas 5,2% das situações.

**Conclusão:**

A saturação venosa de oxigênio femoral não deve ser utilizada em substituição da saturação venosa central de oxigênio. No entanto, os níveis femorais de lactato podem ser utilizados na prática clínica, mas com cautela.

## INTRODUCTION

Plasma lactate levels and central venous oxygen saturation (ScvO_2_) are central hemodynamic parameters in shock management.^(1)^ Hyperlactatemia is commonly present in critically ill patients with acute circulatory failure, indicating abnormal metabolism. Regardless of the mechanism of production, an elevated lactate concentration is a marker of disease severity.^(2,3)^ The ScvO_2_ can provide important information about the balance between oxygen transport and oxygen consumption.^(4)^ In the context of hyperlactatemia, a low ScvO_2_ could indicate inadequate O_2_ delivery in relation to the metabolic demands.^(1,5,6)^

One of the major factors limiting ScvO_2_ assessment in critically ill patients is catheterization of the femoral vein instead of the subclavian or internal jugular vein.^(7,8)^ In many settings, the only deep venous access option is the femoral vein, either because of the unavailability of other puncture sites or because this is an easy access point with no risk of pneumothorax or delay from radiological confirmation, which is otherwise required when access is obtained in the jugular or subclavian veins.^(9)^

A few studies have reported that femoral venous oxygen saturation (SfvO_2_) is not a surrogate for ScvO_2_.^(9-11)^ However, none of these studies has analyzed the agreement between those variables while considering the presence of hypoperfusion signs or clinical decision process impacts. Another interesting question is whether the lactate levels taken from femoral access (LacF) can be used to estimate the arterial lactate (LacA) level. Studies comparing arterial and venous lactate have reported conflicting results.^(12-19)^

Therefore, our objective was to assess whether there is correlation and agreement between the ScvO_2_ and SfvO_2_ and between the LacA and LacF levels in critically ill patients as well as whether this agreement is modified by the presence of hypoperfusion signs. We also aimed to determine whether the use of the SfvO_2_ and LacF level would result in different clinical management.

## METHODS

This prospective, observational study was conducted in four medical-surgical intensive care units (ICU) in two Brazilian university hospitals. The Research Ethics Committee of the *Universidade Federal de São Paulo* approved the study under number 0310/11, and all patients or their legal representatives signed an informed consent form.

The study included patients over 18 years of age who were admitted to the ICU and had both a femoral venous catheter (20cm in length) and a central venous catheter in the subclavian or internal jugular vein. An indwelling arterial catheter was also needed. We excluded pregnant women and patients with a functioning arteriovenous fistula, amputated limbs, venous thrombosis, or barbiturate coma, as well as those who were readmitted to the ICU or who had already been included in the study.

We collected blood samples simultaneously from both the arterial line and distal ports of the central and femoral catheters. The position of the tip of the central venous catheter was confirmed by chest X-ray. The first 5mL of blood drawn from each sample was discarded to prevent dilution. A maximum of six sets were obtained from each patient every 6 hours (T0, T6, T12, T18, T24 and T30). Blood was immediately stored on ice, and all measurements were performed within a maximum of 30 minutes after collection. The samples were assayed using the blood gas analyzer (ABL 700 Radiometer, Copenhagen, Denmark). We excluded all samples with ScvO_2_ or SfvO_2_ > 85% if the patients also had hyperoxia, which was defined by the presence of an oxygen arterial pressure (PaO_2_) > 120mmHg.

We recorded demographic characteristics, comorbidities, and severity scores, namely the Acute Physiologic and Chronic Health Evaluation (APACHE II) score and the Sequential Organ Failure Assessment (SOFA) score. In addition, laboratory data and vasoactive drug doses were recorded at the time of sampling.

We determined the correlation and agreement between the SvcO_2_ and SvfO_2_ and between the LacF and LacA levels, including a pre-specified subgroup analysis in patients with signs of hypoperfusion, which was defined as arterial lactate > 18mg/dL or SvcO_2_ < 70%. We also included a post-hoc subgroup analysis considering those patients under sedation, using mechanical ventilation or using high noradrenaline doses (≥ 0.5µgr/K/min). We assessed the impact of femoral blood samples in clinical management. A board-certified intensivist was asked to recommend clinical interventions based on the SfvO_2_, ScvO_2_ and LacF and LacA levels. The values were presented in a random blinded fashion; the attending intensivist had full access to the patient-specific information.

### Statistical analysis

The sample size was calculated considering the presence of a correlation between SfvO_2_ and ScvO_2_ with r = 0.5 as the null hypothesis, and the alternative hypothesis as a correlation with r = 0.7, using the two-tailed test, an alpha error of 0.05 and 80% power. Based on this, 82 paired samples were necessary.

Continuous variables are reported as the median and interquartile range (IQR) after normality was assessed using the Kolmogorov-Smirnov test. Categorical variables are presented as the absolute values and frequency. We compared the median values of all variables using the Mann-Whitney test. The paired samples were analyzed using the Spearman correlation coefficient (r). We used the Bland-Altman test to describe agreement between the quantitative measurements by constructing limits of agreement. These statistical limits are calculated by using the mean and the standard deviation(s) of the differences between two measurements. The results of the Bland-Altman test are expressed as the bias ± standard deviation (confidence interval 95%), and the confidence interval represented the limits of agreement (LOA).

We used SPSS version 17.0 for Windows (SPSS Inc., Chicago, IL, USA) and GraphPad Prism 5 (GraphPad Software, La Jolla, CA, USA) to perform the statistical analysis. The results with p-values of < 0.05 were considered significant.

## RESULTS

From April 2011 to November 2012, we obtained 107 simultaneous blood samples from 26 patients. The clinical and demographic data are shown in table 1.

**Table 1:** Patient characteristics at baseline

Variables	Results
	(N = 26)
Age (years)	60.5 (54.5 - 69.0)
Gender, male	18 (69.2)
Chronic coexisting conditions	
Cancer	8 (30.8)
Immunosuppression	7 (26.9)
Alcohol use	4 (15.4)
Hypertension	8 (30.8)
Diabetes	3 (11.5)
Coronary artery disease	3 (11.5)
COPD	3 (11.5)
Diagnosis	
Septic shock (abdominal source)	6 (23.1)
Septic shock (pneumonia)	5 (19.2)
Liver transplant	3 (11.5)
Others	12 (46.2)
APACHE II score	22.5 (17.7 - 27.2)
SOFA at admission	9.5 (6.0 - 12.0)
SOFA at inclusion	11.0 (9.0 - 14.2)

COPD - chronic obstructive pulmonary disease; APACHE II - Acute Physiologic and Chronic Health Evaluation; SOFA - Sequential Organ Failure Assessment. Data are presented in numbers (%) and the median (25% - 75%).

**Figure 1:**
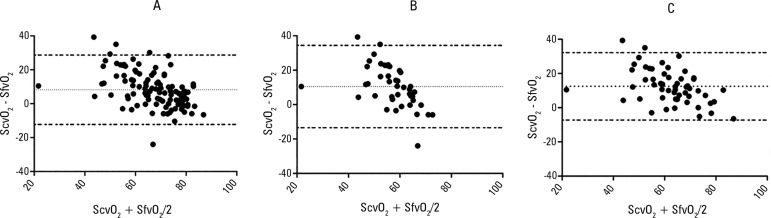
Bland-Altman plots of the difference between ScvO_2_ and SfvO_2_. A) all samples, B) samples with SvcO_2_ < 70%, and C) samples with LacA > 18mmHg. ScvO_2_ - central venous oxygen saturation; SfvO_2_ - femoral venous oxygen saturation; LacA: arterial lactate.

SfvO_2_ values were lower than ScvO_2_ values (63% (53% to 76%) and 72% (66% to 78%), p < 0.0001). Analyzing all 107 pairs of ScvO_2_ and SfvO_2_, we found a moderate correlation between these variables (r = 0.686, p < 0.0001). Bland-Altman analysis resulted in a bias of 8.24 ± 10.44 (95% LOA of -12.23 to 28.70) (Table 2 and Figure 1A).

**Table 2:** Correlation coefficient and agreement for blood taken from different sites - subgroup analysis

Situations	Correlation	p value	Bias	95% LOA
All samples				
SvcO_2_ and SfvO_2_ (N = 107)	0.686	< 0.0001	8.24 ± 10.44	-12.23 to 28.70
LacA and LacF (N = 102)	0.972	< 0.0001	-2.71 ± 9.86	-22.03 to 16.61
SvcO_2_ < 70%				
SvcO_2_ and SfvO_2_ (N = 43)	0.306	0.046	10.50 ± 12.20	-13.42 to 34.42
LacA and LacF (N = 40)	0.940	< 0.0001	-4.54 ± 10.50	25.12 to 16.05
Hyperlactatemia				
SvcO_2_ and SfvO_2_ (N = 54)	0.705	< 0.0001	12.48 ± 10.05	-7.21 to 32.17
LacA and LacF (N = 53)	0.949	< 0.0001	-3.51 ± 13.53	-30.03 to 23.01

LOA - limits of agreement; ScvO_2_ - central venous oxygen saturation; SfvO_2_ - femoral venous oxygen saturation; LacA - arterial lactate; LacF - femoral lactate.

In 43 samples, the ScvO_2_ was below 70%, and the agreement between SfvO_2_ and ScvO_2_ was worse with 10.50 ± 12.20 bias (95% LOA of -13.42 to 34.42) (Figure 1B) and a weak correlation between these variables (r = 0.306, p = 0.046). In the subgroup of patients with arterial hyperlactatemia (54 samples), although the correlation was strong (r = 0.705, p < 0.0001), the bias was large, 12.48 ± 10.05 (95% LOA of -7.21 to 32.17) (Figure 1C). There were similar results when we considered samples with a normal SvcO_2_ and LacA (Table 1S, Figure 1SA and 1SB in the electronic supplementary materials - ESM). Overall, the analysis in patients under sedation, using mechanical ventilation or using high doses of noradrenaline showed similar results with large LOA (Table 1S in the ESM). The clinical agreement between the ScvO_2_ and SfvO_2_ was also inadequate; there were different therapeutic decisions in 22.4% of the situations.

**Figure 2:**
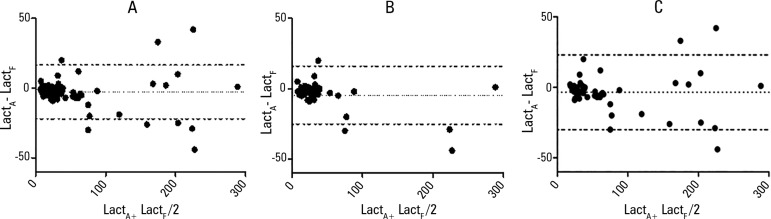
Bland-Altman plots of the difference between the LacA and LacF levels. A) all samples, B) samples with SvcO_2_ < 70%, and C) samples with LacA > 18mmHg. LacA - arterial lactate; LacF - femoral lactate; ScvO_2_ - central venous oxygen saturation.

In the 102 paired lactate samples that we evaluated, the LacF and LacA levels were similar (21.0 (14.0 to 39.0) and 19.5 (12.2 to 39.0), p = 0.299) with a strong correlation between them (r = 0.972, p < 0.001). In the Bland-Altman analysis, there was a bias of -2.71 ± 9.86 (95% LOA of -22.03 to 16.61) (Table 2 and Figure 2A). In the subgroup of patients with low ScvO_2_ levels (n = 40), the correlation was strong (r = 0.940, p < 0.0001) with a bias of -4.54 ± 10.50 and 95% LOA of -25.12 to 16.05 (Figure 2B). Similar findings were observed in the subgroup of patients with arterial hyperlactatemia (53 samples, r = 0.949, p < 0.0001; bias = -3.51 ± 13.53 (95% LOA of -30.03 to 23.01), Figure 2C) and without signs of hypoperfusion (Table 1S and Figure 2SA and 2SB in the ESM) as well in patients under sedation, using mechanical ventilation or using high doses of noradrenaline (Table 1S in the ESM). The clinical agreement between the LacF and LacA levels was good; there was a similar therapeutic decision in 94.8% of the situations.

## DISCUSSION

In this study, we observed that venous blood from the femoral vein is not a reliable substitute for assessing the central venous oxygenation in critically ill patients. Despite a moderate correlation between the SvfO_2_ and ScvO_2_, there is no adequate agreement, and its use resulted in discordant clinical management in a high percentage of the cases. By contrast, the femoral and arterial lactate levels are strongly correlated, and their use led to similar clinical approaches, albeit with a large 95% LOA in the Bland-Altman analysis.

Although the use of ScvO_2_ as a target of resuscitation in septic patients in the emergency department has recently been questioned,^(20-22)^ this remains a useful tool for evaluating the O_2_ demand/supply adequacy in critically ill patients.^(1)^ As many ICU patients only have femoral catheters, it would have been relevant to show the utility of the SfvO_2_. However, our results are in agreement with previously published reports. Van Beest et al.,^(9)^ Davison et al.^(10)^ and Groombridge et al.^(11)^ reported high limits of agreement, suggesting that the SfvO_2_ cannot replace ScvO_2_. However, the last two studies did not have a sample size calculation and were performed on a small number of samples. Although van Beest at al. included a larger number of samples, only 60 were from critically ill patients.^(9)^ In addition, these authors did not analyze the agreement considering subgroups of patients with or without signs of hypoperfusion. We separately evaluated samples from patients with or without tissue hypoperfusion, assessed both by ScvO_2_ and arterial lactate and found that SfvO_2_ did not reliably reflect ScvO_2_, either in patients with abnormal arterial lactate levels or ScvO_2_ or in patients with normal levels. Recently, Zhang et al. also demonstrated wide limits of agreement between the 731 pairs of blood samples collected from an unselected group of 357 critically ill patients. Interestingly, the authors measured the blood flow in the common carotid artery and the femoral artery with Doppler ultrasound in a group of patients. The ratio of common carotid artery flow over femoral artery flow varied widely, suggesting the importance of the blood flow redistribution mechanism.^(23)^

ScvO_2_ is a complex physiologic parameter that is widely used as a resuscitation goal in critically ill patients. Therapeutic interventions may induce strong and eventually divergent effects on the physiologic determinants of oxygen transport (DO_2_) and oxygen consumption (VO_2_) and, thus, on ScvO_2_. For example, ScvO_2_ increases significantly in response to emergency intubation in the majority of septic and non-septic patients.^(24)^ Although SfvO_2_ was significantly correlated with ScvO_2_, the limits of agreement remained large in different interventions tested in our study.

There are several potential reasons for the discrepancy between the venous saturation values. Central venous catheters inserted via the jugular or subclavian veins provide data from cerebral and upper limb oxygen extraction. In situations of physiologic stress, perfusion to kidneys, muscle, and splanchnic regions of the body may be decreased, while flow to the myocardium and brain is relatively preserved. Because of this blood flow redistribution mechanism, the oxygen extraction ratio of the organs and tissues that are drained by the inferior vena cava increased. Thus, ScvO_2_ became higher than SfvO_2_. Because the femoral catheter's tip remains in the iliac vein, the blood collected from this site reflects the oxygen extraction primarily of the pelvis, external genitalia, and lower limbs. Our result aligns with other studies that have compared ScvO_2_ and SfvO_2_ in critically ill patients.^(4,23)^

We could not find previous reports comparing the LacF and LacA levels, although some studies have assessed the reliability of venous lactate as a substitute for the LacA level.^(12-19)^ The authors reported strong correlations between the two values, both venous blood from peripheral veins and from central venous access, but reported inadequate limits of agreement. One study reported inadequate clinical agreement with the peripheral samplings but not with the central line samples.^(12)^ Our contribution to the previous findings was the combination of our evaluation of the femoral venous blood source and the impact of its use in clinical management of the patient. Our findings suggest that the inadequate agreement does not seem to interfere with the clinical decision process.

We found better correlation and agreement between lactate levels of the different sites in comparison to the oxygen saturation values. It is evident that the lactate production and oxygen consumption may increase heterogeneosly in different vascular beds of critically ill patients, leading to different values depending on the place of collection. However, the variability is higher to venous oxygen content than for the lactate. A possible explanation would be a complex metabolism of lactate,^(3)^ whose clearance depends on several passages through the hepatic circulation, while in the case of venous saturation reoxigenation occurs in the lung with each heartbeat.

Our study has some strengths. First, the design was prospective, and the study was conducted in several ICUs with different patient profiles. The study results reinforce previous data with a large number of samples that were exclusively obtained from critically ill patients. We also evaluated, in a blinded fashion, the degree of agreement in clinical management for different sampling sites; our design has not previously been used to evaluate this topic.

However, this study has some limitations. Although the sample size was sufficient, the number of patients was low, and several samples were collected from the same patient. In addition, our patients were in different stages of hemodynamic resuscitation; some patients were in the initial phase with multiple organ dysfunction, and others had already stabilized. However, this heterogeneity does not necessarily jeopardize the presented results. A further limitation is that we only included patients who had deep venous accesses in both the jugular or the subclavian and the femoral veins, which may have caused sampling bias.

## CONCLUSION

In conclusion, femoral venous oxygen saturation should not be used as a replacement for central venous oxygen saturation. However, the strong correlation and satisfactory clinical agreement between the femoral lactate and arterial lactate levels allows for the use of the femoral lactate level in clinical practice, albeit with caution because the limits of agreement were wide.
