# Supplementary material for: Is venous blood drawn from femoral access adequate to estimate the central venous oxygen saturation and arterial lactate levels in critically ill patients?
Source: Rev Bras Ter Intensiva. 2015 Oct-Dec;27(4):340–6. doi: 10.5935/0103-507X.20150058 (PMC4738819; doi:10.5935/0103-507X.20150058)
Supplement: Supplementary file 1 [file rbti-27-04-0340-suppl01.pdf]

Is venous blood drawn from femoral access adequate to estimate the central venous oxygen saturation and arterial lactate levels in critically ill patients?

O sangue venoso coletado do acesso femoral é adequado para estimar a saturação venosa central de oxigênio e os níveis de lactato arterial em pacientes graves?

Yara Nishiyama Marti[1], Flávio Geraldo Rezende de Freitas[1], Rodrigo Palácio de Azevedo[1], Milena Leão[1], Antônio Tonete Bafi[1], Flavia Ribeiro Machado[1]

Additional results

Table 1S - Correlation coefficient and agreement for blood taken from different sites - subgroup analysis

Situations	Correlation	p value	Bias	95% LOA
$ScvO_2 \geq 70\%$				
$SvcO_2$ and $SfvO_2$ (N = 64)	0.380	0.002	6.71 ± 8.85	-10.63 to 24.05
LacA and LacF (N = 62)	0.964	< 0.0001	-1.53 ± 9.32	-19.80 to 16.73
Lactate ≤ 18 mg/dL				
$SvcO_2$ and $SfvO_2$ (N = 50)	0.461	0.001	3.93 ± 9.30	-14.30 to 22.15
LacA and LacF (N = 49)	0.826	< 0.0001	-1.85 ± 2.18	-6.13 to 2.44
High dose noradrenaline				
$SvcO_2$ and $SfvO_2$ (N = 39)	0.809	< 0.0001	16.19 ± 9.51	-2.45 to 34.84
LacA and LacF (N = 35)	0.969	< 0.0001	-3.51 ± 15.84	-34.55 to 27.52
Without high dose noradrenaline				
$SvcO_2$ and $SfvO_2$ (N = 68)	0.724	< 0.0001	3.67 ± 7.94	-11.90 to 19.25
LacA and LacF (N = 67)	0.933	< 0.0001	-2.29 ± 4.36	-10.84 to 6.25
Under sedation				
$SvcO_2$ and $SfvO_2$ (N = 41)	0.721	< 0.0001	11.19 ± 10.44	-9.28 to 31.65
LacA and LacF (N = 63)	0.972	< 0.0001	-2.09 ± 14.44	-30.39 to 26.21
No sedation				
$SvcO_2$ and $SfvO_2$ (N = 66)	0.694	< 0.0001	6.40 ± 10.09	-13.37 to 26.18
LacA and LacF (N = 39)	0.953	< 0.0001	-3.09 ± 5.49	-13.86 to 7.66
Mechanical ventilation				
$SvcO_2$ and $SfvO_2$ (N = 99)	0.705	< 0.0001	8.99 ± 10.38	-11.35 - 29.33
LacA and LacF (N = 94)	0.974	< 0.0001	-2.78 ± 10.26	-22.89 to 17.33
Spontaneous ventilation				
$SvcO_2$ and $SfvO_2$ (N = 8)	0.357	0.385	-1.10 ± 5.93	-12.74 to 10.54
LacA and LacF (N = 8)	0.798	< 0.0001	-1.87 ± 1.64	-5.09 - 1.34

LOA - limits of agreement; $ScvO_2$ - central venous oxygen saturation; $SfvO_2$ - femoral venous oxygen saturation; LacA - arterial lactate; LacF - femoral lactate. High dose of noradrenaline was defined as ≥ 0.5 μg/kg/min.

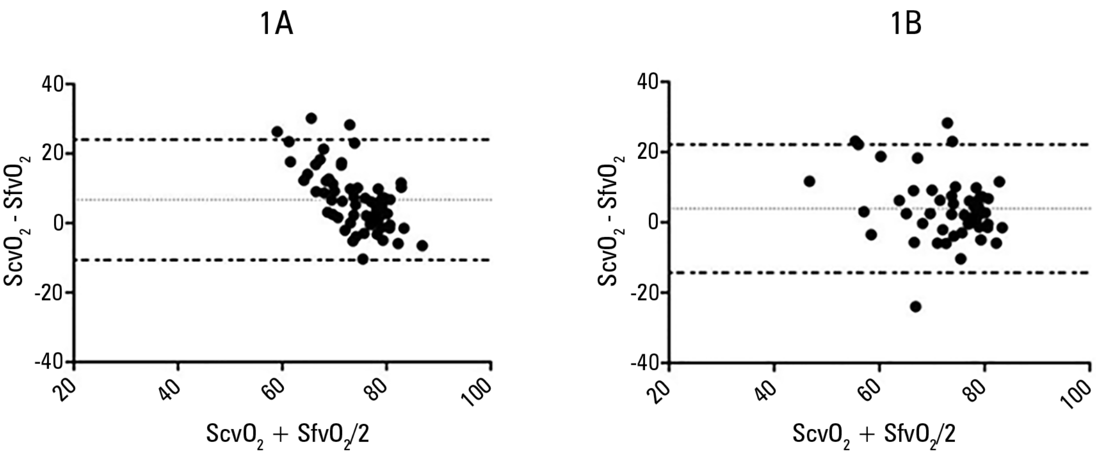

Figure 1S - Bland-Altman plots of the difference between $ScvO_2$ and $SfvO_2$ when $SvcO_2$ was ≥ 70% (1A) and when the lactate level was ≤ 18mmHg (1B). $ScvO_2$ - central venous oxygen saturation; $SfvO_2$ - femoral venous oxygen saturation.

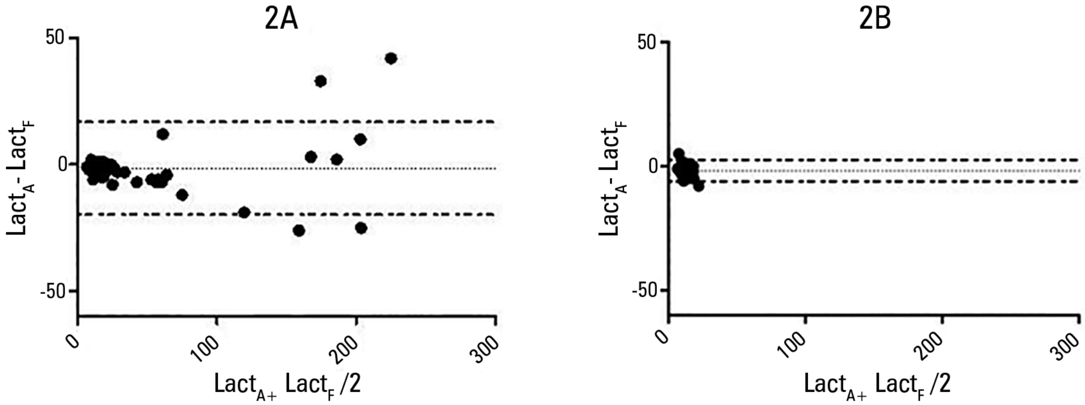

Figure 2S - Bland-Altman plots of the difference between LacA and LacF when $SvcO_2$ was ≥ 70% (2A) and when the lactate level was ≤ 18mmHg (2B). LacA - arterial lactate; LacF - femoral lactate.
